# Development and optimization of a new competitive ELISA using recombinant (rPSA D15 and rCag11) antigens for the detection of *Helicobacter pylori* infection

**DOI:** 10.1371/journal.pone.0317227

**Published:** 2025-01-15

**Authors:** Biniam Moges Eskeziyaw, Naomi Maina, Rebecca Waihenya, Matthew Mutinda Munyao, Tonny Teya Nyandwaro, Shingo Inoue, Samson Muuo Nzou

**Affiliations:** 1 Pan African University for Basic Science, Technology and Invocation (PAUSTI), Nairobi, Kenya; 2 Department of Biotechnology, Debre Berhan University, Debre Berhan, Ethiopia; 3 Department of Biochemistry, Jomo Kenyatta University of Agriculture and Technology, (JKUAT), Juja, Kenya; 4 Department of Zoology, Jomo Kenyatta University of Agriculture and Technology (JKUAT), Juja, Kenya; 5 Kenya Medical Research Institute, Centre for Microbiology Research, Nairobi, Kenya; 6 Nagasaki University Institute of Tropical Medicine-Kenya Medical Research Institute Project, Nairobi, Kenya; Abdul Wali Khan University Mardan, PAKISTAN

## Abstract

*H*. *pylori (Hp)* is highly causative agent of chronic gastritis, gastric cancer and human death worldwide. To address the challenge of *H*. *pylori* infection, numerous immunological assays have been developed for its diagnosis and management. However, the limited availability of these assays in certain laboratories, coupled with their high cost, inconsistent specificity, and sensitivity, has hampered their widespread adoption, particularly in developing countries where *H*. *pylori* infection is prevalent. Therefore, this study aimed to develop and validate a competitive enzyme-linked immunosorbent assay (cELISA) assay for detecting *H*. *pylori* infections by targeting the Protective Surface Antigen (PSA) and Cytotoxic-Associated Gene Pathogenesis Island (Cag11) proteins in *H*. *pylori* stool antigen sample. In the current study, the optimal conditions including the dilution of anti-rPSA D15 and anti-rCag11 antibodies at 1:1000, coating antigens (rPSA D15 and rCag11) at a concentration of 1 μg/well, the dilution of HRP-labelled antibody at 1:5000 and *H*. *pylori* stool antigen dilution at 1:1000 with a 1hour incubation and color development time of 30 minutes for cELISA were determined using an ELISA checkerboard titration assay. Based on the optimized conditions, novel rPSA D15-cELISA and rCag11-cELISA assays with a respective optimum cut-off value of 20.80% PI and 24.16% PI were developed. According to the receiver operating characteristic (ROC) curve analysis on the diagnostic performance of the newly developed rPSA D15-cELISA and rCag11-cELISA assays using 60 clinical *H*. *pylori* stool samples, the rPSA D15-cELISA test assay established an optimum cut-off point of 20.80% with sensitivity and specificity of 90% (95% confidence of interval (CI) 74.38–96.54), Area under the curve (AUC) of 0.9556 (95% CI = 0.896–1.000) and P value <0.0001. Similarly, the rCag11-cELISA assay revealed optimum cut-off value of 24.16% with sensitivity of 93.33% (95% CI 78.68–98.82), specificity of 90% (95% CI 74.38–96.54), AUC of 0.986 (95% CI = 0.967–1.000) and P <0.0001. Furthermore, the reproducibility assay coefficients of variation (CV) of the newly developed rPSA D15-cELISA and rCag11-cELISA assay were less than 10%, indicating that the two cELISA assays exhibits excellent reproducibility and reliability. To validate their clinical diagnostic application, the comparative study results of rPSA D15-cELISA and rCag11-cELISA showed a high agreement (k = 0.766 and 0.799) with the commercially available *H*. *pylori* antigen test immunochromatographic kit and more accurate than the reference kit by detecting stool antigen of *H*. *pylori* strain, indicating it is promising for clinical testing. In conclusion, these results indicated that the newly developed rPSA D15-cELISA and rCag11-cELISA *H*. *pylori* stool antigen test assays were a potential reliable and a clinically useful assay for rapid, specifically, sensitively and accurately diagnosis and large-scale epidemiological investigation of *H*. *pylori* infection.

## 1. Introduction

*Helicobacter pylori* infection is one of the most prevalent bacterial infections among humans, impacting over 50% of the global population [1] and cause for over 700,000 fatalities each year across the globe [2]. *H*. *pylori* infection is associated with a range of serious health issues, including chronic gastritis, peptic ulcers, gastric cancer, and mucosa-associated lymphoid tissue (MALT) lymphoma, as well as various extra-gastric disorders that can lead to mortality on a global scale. Consequently, it is essential to implement early and precise diagnostic measures, maintain appropriate follow-up care, and explore alternative treatment options by identifying both diagnostic and therapeutic targets [3].

Throughout a year, various diagnostic (both invasive and non- invasive) assays have been developed for the diagnosis and management of *H*. *pylori* infection [4]. Currently, *H*. *pylori* is primarily identified using non-invasive tests like PCR, urea breath tests, and enzyme immunoassays. In contrast, invasive testing necessitates an upper gastrointestinal endoscopy. Additionally, techniques like urea breath tests and PCR require costly equipment and skilled technicians, making them labour-intensive and time-consuming to perform [5].

The adoption of non-invasive ELISA testing for *H*. *pylori* has been highly recommended due to its speed, affordability, ease of use, ability to process large volumes, and overall patient-friendliness when compared to both invasive and other non-invasive tests. However, the limited availability of these assays in certain laboratories, coupled with their high cost, inconsistent specificity, and sensitivity, has hampered their widespread adoption, particularly in developing countries where *H*. *pylori* infection is prevalent [6,7]. Therefore, this study aims to create a rapid, cost-effective, and precise competitive enzyme-linked immunosorbent assay (cELISA) for detecting *H*. *pylori* infections by targeting conserved antigens to address the various genotypes of *H*. *pylori*.

The current study reports the development and validation of novel rPSA D15-cELISA and rCag11-cELISA diagnostic assay using a rPSA D15 and rCag11 proteins as coating antigen, *H*. *pylori* stool antigen as detecting antigen and anti-rPSA D15 and anti-rCag11 polyclonal antibodies, along with HRP-conjugated antibodies in a competitive format to detect and quantify *H*. *pylori* antigens in stool samples. Fortunately, the findings of this study revealed that the newly developed rPSA D15-cELISA and rCag11-cELISA assays were a potential reliable and a clinically useful assay for rapid, specifically, sensitively and accurately diagnosis and large-scale epidemiological investigation of *H*. *pylori* infection disease in Africa setting and world at large.

## 2. Materials and methods

### 2.1. Ethics statement

In this study, a scientific and ethical approval was obtained from the Mount Kenya University Internal Ethical Review Committee (Ref No: MKU/ISERC/3558 and Approval No: 2602). During human stool samples collection, a written consent was obtained from participants.

For polyclonal antibody production, animal (New Zealand White rabbits 3-months old, approximately 2.0 kg) studies were carried out in strict accordance with the guidelines of the KEMRI Animal care and use committee. The protocol was approved by the KEMRI Animal care and use committee (KEMRI ACUC) (approval number KEMRI ACUC/01.07.2023).

The experimental rabbits were subjected to a controlled sacrifice procedure using a carbon dioxide (CO2) chamber to ensure effective anaesthesia and minimize distress. Initially, the rabbits were placed in the CO2 chamber, where CO2 (approximately 20% of the chamber volume per minute) was gradually introduced to induce anaesthesia and subsequent unconsciousness and painless euthanized. The rabbits were allowed to remain in the chamber until respiratory arrest was clearly observed. This process ensured a swift and painless method of sacrifice, minimizing any suffering associated with the procedure. Following confirmation of adequate anaesthesia, whole blood was collected through cardiac puncture, performed via surgical intervention to ensure minimal trauma. Throughout the process, efforts to alleviate suffering were prioritized, including careful monitoring of the rabbit’s responses and ensuring that the CO2 exposure was adjusted to minimize any potential discomfort, thereby adhering to ethical standards while achieving the objectives of the study.

### 2.2. Sample collection and processing

In this study, we followed rigorous protocols for the collection and processing of *H*. *pylori* stool antigen samples for the ELISA test to ensure accuracy and reliability. From April 10 to July 21, 2024, we collected approximately 60 fresh stool samples (30 from *H*. *pylori* positive patients and 30 from negative patients) from individuals who provided written consent. The H. pylori status of these patients was confirmed using a commercially available immunochromatographic kit (SAFECARE BIO-TECH, Beijing, China) at the OASIS Laboratory Center. To maintain the integrity of the samples, they were immediately stored at 4°C until processing at the Molecular Biology and Biotechnology Lab at PAUSTI.

Within 24 hours of collection, each stool sample was thoroughly homogenized with a sterile buffer solution to prepare a uniform suspension. Subsequently, the suspension was centrifuged to separate solids from the liquid phase, and the supernatant was carefully collected. This supernatant was then aliquoted into sterile tubes and transported to Pan-African Hub for Infectious Disease Research Laboratory, Kenya Medical Research Institute (KEMRI) for further ELISA processes and frozen at-20 °C until the ELISA test performed. Prior to the ELISA test, the frozen samples were thawed and gently mixed to ensure homogeneity.

These *H*. *pylori* stool antigens samples were used to evaluate the analytical and diagnostic sensitivity, specificity and reproducibility of the newly developed rPSA D15-cELISA and rCag11-cELISA assay resulted in a statistical power for a likely diagnostic sensitivity or diagnostic specificity of 95% confidence within 5% margin of error.

### 2.3. Cloning, expression and purification of recombinant (rPSA D15 and rCag11) antigen

Based on the results of previous immunoinformatic study [8], we successfully expressed recombinant (~24kDa rPSA D15 and ~21 kDa rCag11) antigens, derived from *H*. *pylori* in the *E*. *coli BL21(DE3)* bacterial system. These antigens were purified using high-affinity nickel-nitrilotriacetic-acid (Ni NTA) resin column chromatography and their purity and immunoreactivity were evaluated by SDS-PAGE and western blotting in our previous study (unpublished data). The purified recombinant (rPSA D15 and rCag11) proteins were subsequently utilized as immunogen to generate anti-rPSA D15 and anti-rCag11 polyclonal antibodies. Additionally, they served as the coating antigens in the newly developed rPSA D15-cELISA and rPSA D15-cELISA assays.

### 2.4. Production and characterization of antibody against recombinant antigens

For anti-rPSA D15 and anti-rCag11 polyclonal antibody production, five New Zealand White female rabbits (12-weeks-old) were purchased from FARMWORX (Nairobi, Kenya) and acclimated for 14 days in a controlled environment condition with complete free access to the standard rabbit food and water at the animal facility of the Kenya Medical Research Institute.

After allowing the rabbits to adapt to their new environment for two weeks, 1 ml of blood sample (pre-immunization sera) was taken from the marginal vein of the rabbit’s ears for use as a baseline negative control, prior to each immunization. For the 1^st^ injection, ~2ml of each purified rPSA D15 and rCag11 antigens (500 μg/injection) were emulsified with 2ml of Freund’s Complete Adjuvant (FCA) (Sigma, Japan) (1:1 v/v) and injected subcutaneously into the hind leg of each rabbit at two different sites. At 14, 28 and 42 days after the first immunization, the second, third and fourth subsequent booster injection were administered using the same amount of purified rPSA D15 and rCag11 antigens and Freund’s incomplete adjuvant. Similarly, the negative control rabbit was immunized with four times with two weeks interval. A control rabbit underwent a similar procedure, but with a PBS (500 μl/injection) instead of the immunogen.

After immunization, rabbits’ blood (~2ml) was collected from marginal ear vein on d 0, 14, 28, 42. Whole blood was also collected by cardiac puncture on d 56 and allowed to clot at room temperature for 30 minutes at room temperature. Then, the clotted blood was centrifuged at 5000 rpm for 10 min at 4°C and the clear supernatant phase (PcAb) was carefully collected and stored at -30°C until used. The concentration of IgG was determined by BCA assay and stored at −30°C. The serum titer level of the purified rPSA D15 and rCag11antigens-specific antibodies at each group were analysed using indirect Enzyme-Linked Immunosorbent Assay (iELISA) using the purified rPSA D15 and rCag11 proteins as a coating antigen following the manufacture instruction.

### 2.5. Development and optimization of competitive enzyme linked immunosorbent assay

In the current study, the optimized novel rPSA D15-cELISA and rCag11-cELISA assay were developed using the optimized rPSA D15/rCag11 coting antigens, HRP-conjugated mAb and anti-rPSA D15/ rCag11 pAb. Briefly, different amounts of the rPSA D15/rCag11 antigen (250, 500, 750, 1000, 2000, 3000 ng/well) were coated into the 96-well plates, then diluted HRP-conjugated mAb (1:5000 dilution in PBS-Tween, with 0.1% Blockace) were tested. Finally, the optimal amount of coating antigen and HRP-conjugated mAb with high OD_492_nm Percentage of inhibition (PI%) were selected. Secondly, the optimal dilution ratio of tested *H*. *pylori* stool antigen sample and anti-rPSA D15 and anti-rCag11 pAb sera were determined. For this, two separate positive and negative diluted stool and anti-rPSA D15 and anti-rCag11 pAb serum samples (neat, 1:10, 1:100, 1:1000, 1:2000 and 1:4000, 1:8000, 1:16000, 1:32000, 1:64000, 1:128000 and 1:256000 dilution in PBS-Tween, with 10% Blockace) were tested. The optimal *H*. *pylori* stool antigen and serum sample dilution was determined according to the highest PI% values). Finally, the times of incubation and color reaction after the addition of tetramethylbenzidine (TMB) were separately optimized. The incubation times of the mixtures containing the HRP-conjugate mAb and the positive or negative *H*. *pylori* stool antigen and sera with coated rPSA D15 and rCag11 antigens were tested. After incubation, TMB was added to color and tested after 10, 15, and 20 min. The smallest ratio of OD_494_nm (highest PI%) values between the positive and negative sera was selected as the optimal incubation and colorimetric reaction times.

After optimizing the above parameters, the standard procedure of the newly developed rPSA D15-cELISA and rCag11-cELISA assays were performed as follows. (1) The 96-well ELISA plate (Nunc, Thermo Fisher Scientific) was coated with the optimal amount (1000ng/100 μL /well) of recombinant (rPSA D15/rCag11) antigens except for Blank (Blank: no reagent) and incubated overnight at 4°C. (2) The plate was blocked with 100 μl of original concentration Blockace at RT for 1 h. (3) After washed 3x with PBST, 1h pre-incubated 100 μL/well of testing mixtures containing the optimal dilutions (1000x diluted) of *H*. *pylori* stool antigen samples and anti-rPSA D15/rCag11 pAb, was added in all wells except for Blank (PBS-Tween), then incubated for optimal condition (37 °C for 1h). (4) After the plate was washed again in the same way, 100 μl/well of optimal diluted (5,000x diluted) goat anti-rabbit mAb-HRP Conjugate (American Qualex, USA) was added in all wells except for Blank (Blank: PBS-Tween) and incubated for optimal condition (37 °C for 1h). Followed by OPD (Sigma Chemical, St. Louis, MO, USA) (100 μL/well) was added and incubated for optimal times(30min) at RT. (5) Finally, 3 M H2 SO4 (100 μL/well) was used to stop the colorimetric reaction, and the OD_492_nm values were read using an auto mated ELISA plate reader (Bio-Rad, USA) and analysis sample serum OD/Negative control serum OD ratio (P/N ratio); P/N Ratio = (Mean of OD of Sample-Mean OD of Blank)/(Mean of NC-Mean of Blank) and Percent of inhibition of test serum using the formula: Percent inhibition (PI%) of test serum = (1 –OD_492_nm of unknown sample ÷ OD_492_nm of negative sample mean) X 100% [9].

To determine the cut-off point of the newly developed rPSA D15-cELISA and rCag11-cELISA assay, a total of 30 stool samples that were negative for *H*.*pylori* were tested using the rPSA D15-cELISA and rCag11-cELISA assays developed in this study, with the cut-off value for positive results calculated based on the mean PI of 30 negative stool plus 3 standard deviations (CoV = mean PI of negative sample + 3SD) [10] with percentage of specificity and sensitivity reached the maximum value through ROC curve analysis. The result was recognized as positive and negative sample if the PI% ≥ cut off value and PI%< cut-off vale, respectively.

### 2.6. Evaluation of analytical sensitivity and specificity of the developed cELISA assays

The smallest amount (analytical sensitivity) of the antigen in a stool sample that can be accurately detected by the newly developed rPSA D15-cELISA and rCag11-ELISA were determined by testing twofold serial dilutions (neat, 1:10, 1:100, 1:1000, 1:2000 and 1:4000, 1:8000, 1:16000, 1:32000, 1:64000, 1:128000 and 1:256000) of two *H*. *pylori* positive stool sample (confirmed by commercial *H*. *pylori* antigen test immunochromatography kit), standard antigen (known concentration as PC) and dilute buffer samples (NC) were tested using the newly developed rPSA D15-cELISA and rCag11-ELISA assay following the procedure as described above. To quantify the lowest limit of detection of the newly designed rPSA D15-cELISA and rCag11-assay, standard curve using the known concentrated standard antigen sample and the corresponding concentration of the target stool sample were generated and the lowest antigen titter (ng/ml) with a value higher than the cut-off was set as the analytical sensitivity level of the cELISA assays developed in this study. The LOB (Limit of Blank) and the absorbance of LOD (Limit of Detection) were calculated for rPSA D15-cELISA and rCag11-ELISA using following formula; LOB = Mean Blank+1.645(SD Blank); Ab LOD = Mean Blank+3 SD Blank [11].

The analytical specificity of the rPSA D15-cELISA and rCag11-ELISA assay were determined by testing the cross-reactivity of whole cell antigens of pathogenic bacterial (ESKAPE) including Staphylococcus aureus, Klebsiella pneumoniae, Acinetobacter baumannii, Pseudomonas aeruginosa, and Enterobacter species with anti-rPSA D15 and anti-rCag11rabbit purified serum using rPSA D15-cELISA and rCag11-ELISA developed in this study following the procedure as described above.

### 2.7. Determination of diagnostic sensitivity and specificity of the developed cELISA assays

The diagnostic sensitivity of the newly developed rPSA D15-cELISA and rCag11-ELISA were determined by testing 30 *H*. *pylori* positive clinical stool samples (confirmed by the commercial *H*. *pylori* antigen test immunochromatography kit) using rPSA D15-cELISA and rCag11-ELISA assay. Referring the calculated cut-off value, the positive result (PI% values above cut-off value) and negative result (PI% values below cut-off value) were discriminated and the percent of sensitivity of the designed rPSA D15-cELISA and rCag11-ELISA assay were determined in percent using the formula: sensitivity % = (TP/ (TP + FN)) × 100% [12,13].

The diagnostic specificity of the rPSA D15-cELISA and rCag11-ELISA assay were determined by testing 30 *H*. *pylori* negative clinical stool samples (human stool spiked with ESKAPE bacterial whole cell antigen) to confirm their negative *H*. *pylori* status using rPSA D15-cELISA and rCag11-ELISA assay. Referring the calculated cut-off value, the positive result (PI% values above cut-off value) and negative result(PI% values below cut-off value) were discriminated and the percent of specificity of the designed rPSA D15-cELISA and rCag11-ELISA assay were determined in percent using the formula: specificity = (TN/ TN-FP) X100% [14].

Furthermore, the optimal cut-off vale with best discrimination between positive and negative samples and resulting in the best combination of diagnostic sensitivity and specificity of the newly developed rPSA D15-cELISA and rCag11-ELISA assay were determined by receiver-operating characteristic (ROC) curve [plots of sensitivity against (1-specificity)] analysis using GraphPad Prism 10.2.3 software (https://www.graphpad.com/)(San Diego, California, USA).

### 2.8. Determination of the reproducibility of the newly developed cELISA assays

To determine the precision of the rPSA D15-cELISA and rCag11-cELISA assays, three separate *H*. *pylori* positive clinical stool samples were tested on the same ELISA plate in triplicate using rPSA D15-cELISA and rCag11-cELISA assay. The percentage coefficients of variation (%CV) each sample was calculated using the following formula: %CV = (SD of PI _replicate_/Mean _replicate_) X 100. Average of each sample %CVs was reported as the reproducibility CV (CV _intra-assay_) [15]. The intra-assay repeatability of the rPSA D15-cELISA and rCag11-cELISA assay were reported as acceptable where a CV _intra-assay_ value were < 10% [16].

### 2.9. Comparisons of the newly developed *Hp*-cELISA with the commercially *H*. *pylori* diagnostic kit

To compare and correlate the results between the newly developed rPSA D15-cELISA and rCag11-ELISA assays and the available commercial *H*. *pylori* antigen test immunochromatography kit, a total of 60 stool samples (30 *H*. *pylori* positive and 30 negative) were tested by both immunodiagnostic assays following the procedure as described above [17]. Agreement rate between the results obtained using the newly developed rPSA D15-cELISA and rCag11-ELISA assay and results obtained using the commercial *H*. *pylori* rapid immunochromatography kit was determined based on the Cohen Kappa coefficient value (K) using the following formula; $\text{K}=\frac{PA\;or\;Po-Pe}{1-Pe}$ and Where, PA- Percent of agreement and Po- Relative agreement. The agreement rate was reported; Kappa < 0: No agreement; 0.00≤ Kappa value ≤ 0.20: Slight agreement; 0.21 ≤ Kappa value ≤ 0.40: Fair agreement; 0.41≤ Kappa value ≤ 0.60: Moderate agreement; 0.61 ≤ Kappa value ≤ 0.80: Substantial agreement; 0.81≤ Kappa value ≥1.00:Almost perfect(excellent) agreement [18]. Samples yielding inconsistent results were tested by culture as a reference test assay. The coincidence rate (CR) was calculated using the formula CR (%) = [(Total number of stool samples-Number of inconsistent samples) / Total number of stool samples×100%] [19].

### 2.10. Statistical analysis

In the present study, the experimental data were presented as means and standard deviations (SD) with the formula: mean ± SD. The cut-of vale (CoV) of the newly developed rCag11-ELISA and rPSA D15-ELISA were determined using a formula: CoV = PI% mean of OD_492_ nm value of the negative sample + 3SD), with samples were considered positive and negative, if the PI% value was above and below the CoV respectively. The student’s t-test was used to analysed the statistically significant of the difference between the reactivity (ODs value) of known *H*. *pylori*-positive and negative human sera in ELISA using GraphPad Prism 10.2.3 software (https://www.graphpad.com/)(San Diego, California, USA) with a p value< 0.05 and P<0.01 being considered statistically significant and extremely significant, respectively.

The specificity and sensitivity of the newly developed rPSA D15-cELISA and rCag11-cELISA assay were calculated according to the following formulas: specificity (%) = [true negative numbers/ (true negative numbers + false positive numbers)] × 100%: analytical sensitivity (%) = [true positive numbers/ (true positive numbers + false negative numbers)] × 100%. In addition, Negative Predictive Value (NPV), Positive Predictive Value (PPV), and 95% confidence interval (CI) were determined using MedCalc version 12.7. (https://www.medcalc.org/). The optimal cut-off vale resulting in the best combination of diagnostic sensitivity and specificity of the rPSA D15-cELISA and rCag11-ELISA assay were determined using AUC-ROC (Area Under the Receiver Operating Characteristic curve) curve analysis by plotted as True Positive Rate (TPR) versus False Positive Rate (FPR) using GraphPad Prism 10.2.3 software (https://www.graphpad.com/)(San Diego, California, USA).

The repeatability of the rPSA D15-cELISA and rCag11-cELISA assay was assessed using a formula’s = (SD/mean) X 100%, where a CV _intra-assay_ < 10% was considered an acceptable repeatability level for the assay.

Agreements (concordance) between the newly developed rPSA D15-ELISA and rCag11-ELISA and commercial available *H*. *pylori* antigen test immunochromatographic assay kit (reference kit) were estimated using the Kappa coefficient formula κ = (PA-Pe)/1-Pe) and using online available GraphPad software (http://graphpad.com/quickcalcs/kappa1/); where Kappa value ≤0.40 indicated poor agreement, 0.40<Kappa value≤0.6 indicated moderate agreement, 0.6<Kappa value≤0.75 indicated high agreement, and Kappa value>0.75 indicated excellent agreement.

## 3. Results

### 3.1. Cloning, expression and purification of recombinant (rPSA D15 and rCag11) antigens

In our previous study [8], the recombinant (~24k Da rPSA D15 and ~21 kDa rCag11) antigens of *H*. *pylori* were designed and expressed in *E*. *coli BL21*(DE3) bacterial system and purified using a high-affinity nickel-nitrilotriacetic-acid (Ni NTA) resin column chromatography. The purified recombinant proteins were used as the immunizing antigen for production of anti-rPSA D15 and anti-rCag11 pAb and as the coating antigen for the rPSA D15-Cag11 and rPSA D15-cELISA assays developed in this study.

### 3.2. Production and characterization of polyclonal antibody against recombinant antigens

**Fig 1:**
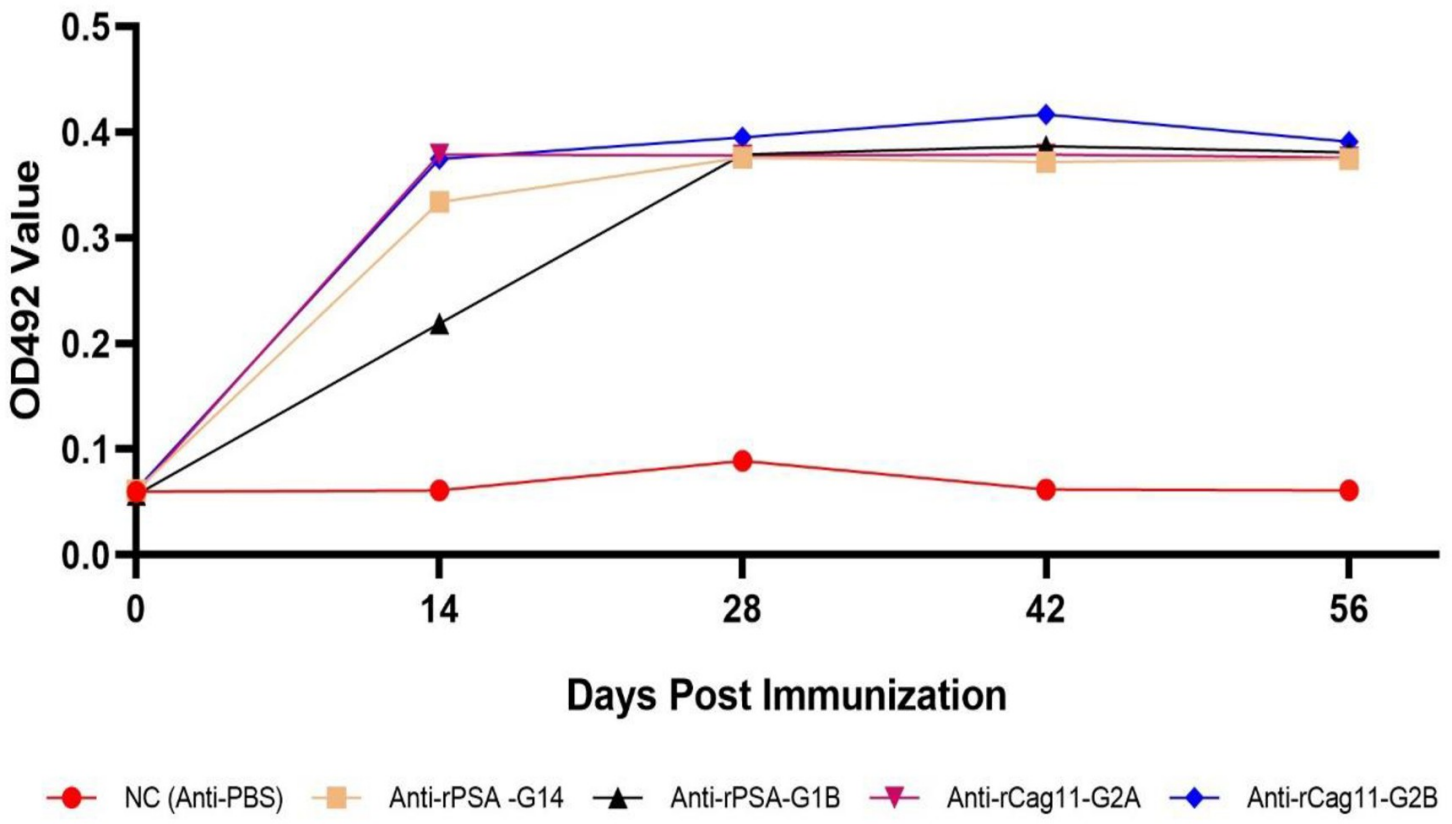
Kinetics of antibody responses against rPSA D15 and rCag11 antigens in rabbits on ELISA assay.

To evaluate the ability of the purified recombinant (rPSA D15 and rCag11) protein to induce host specific antibody responses, 1000 diluted serum from four New Zealand white rabbits (12 weeks old age) immunized with rPSA D15 and rCag11 antigens, serum from one rabbit immunized with PBS and pre-immunized serum were examined via iELISA assay. The result showed that rabbits immunized with rPSA D15 and rCag11 developed a strong anti-rPSA D15 and anti-rCag11 antibodies immune response, as measured by iELISA. The levels of anti-rPSA D15 and anti-rCag11 antibodies increased rapidly in the first 14–28 days and then stabilized at a high level until the end of the 56-day period. In contrast, rabbits that were not immunized with antigens (pre-immunized and PBS-immunized) did not produce any antibodies against rPSA D15 and rCag11 antigens, indicating that the immunization was effective in inducing a targeted immune response against the purified immunodominant *H*. *pylori* antigens (Fig 1). The results suggest that both the expressed and purified rPSA D15 and rCag11 antigens have a high potential to stimulate a strong immune response in rabbits, as evidenced by a significant increase in anti-rPSA D15 and anti-rCag11 antibody titers. This difference was statistically significant (p < 0.001) compared to the pre-immunized and PBS-immunized rabbits. Therefore, these antigens and antibodies can be used to develop an immunodiagnostic assay for the detection of *H*. *pylori* infection.

### 3.3. Development and optimization of rPSA D15-cELISA and rCag11-cELISA assays

In the process of developing and validating a new rPSA D15-cELISA and rCag11-cELISA assay for detecting *H*. *pylori* infection, several crucial parameters were optimized. These included testing various concentrations of coating antigens, determining the best dilution for primary antibodies (anti-rPSA D15 and anti-rCag11), identifying the optimal amount of testing antigens, adjusting the dilutions of HRP-IgG antibodies, and fine-tuning the incubation and color development times. This experimental optimization was carried out using a checkerboard titration assay following the manufacturer’s instructions.

The results from the checkerboard assay indicated that the ideal concentrations for the rPSA D15 and rCag11 coating antigens were 1μg/well, combined with a 1:5000 dilution of the HRP-conjugated secondary antibody. This setup provided high PI% of 52.48(rPSA D15-cELISA) and 59.62%(rCag11-cELISA) for *H*. *pylori* positive stool samples, while the PI% for *H*. *pylori* negative stool samples were significantly lower at 12.84% and 6.082%, respectively. These findings suggest that the coating antigens were at the optimal concentration for effectively capturing the antibodies with minimal non-specific binding (Table 1).

**Table 1:** Optimized amount of rPSA D15 and rCag1 coating antigens and dilution of enzyme-conjugated secondary antibody using the cELISA checkerboard titration assay.

Coating Antigen	HRP-Conjugated mAb (μg/well)	OD_492_ PI% value at different con of coating antigen (μg/well)					
		3.0	2.0	1.0	0.75	0.5	0.25
rCag11	1:5000	46.445	48.332	59.622	50.322	46.273	45.587
	1:5000	20.024	6.900	12.840	8.963	17.677	16.503
rPSA D15	1:5000	39.891	50.734	52.484	50.288	40.714	36.013
	1:5000	8.678	30.979	6.082	12.520	7.006	9.390

*The PI ratio was calculated using the formula PI (%) = [1- (OD_492nm_ of HpSA sample / OD_492nm_ of negative HpSA)] × 100%.

Furthermore, the optimal dilution of the tested *H*. *pylori* stool antigen sample with optimum diluted anti-rPSA D15 and anti-rCag11 antibodies was established at 1:1000 (Fig 3) based on the different dilutions of two positive *H*. *pylori* stool sample that produced the highest PI% of 59.828% for rCag11-cELISA and 63.568% for rPSA D15-cELISA (Table 2). Moreover, the best incubation period for the *H*. *pylori* stool antigen, the primary antibodies (anti-rPSA D15/rCag11), and the HRP-conjugated antibody mixture was determined to be 1hour, while the optimal time for the colorimetric reaction was found to be 15 minutes.

**Table 2:** The optimized *H*. *pylori* stool antigen (HpSA) dilution for rPSA D15- cELISA and rCag11-cELISA assay.

HpSA Dilution	OD_492nm_ value at different dilution of *HpSA* sample		PI Value (%)	
	rCag11	rPSA D15	rCag11	rPSA D15
Original	1.457	1.551	51.313	49.087
1:10	1.626	1.590	46.513	47.749
1:100	1.415	1.614	53.754	46.925
1:1000	1.238	1.129	59.828	63.568
1:2000	1.898	1.793	37.180	40.783
1:4000	1.922	2.007	36.356	33.439
1:8000	1.858	2.239	38.552	25.478
1:16000	2.176	2.324	27.640	22.562
1:32000	2.334	2.753	22.218	7.841
1:64000	2.599	2.776	13.125	7.051
1:128000	2.825	2.89	5.370	3.139
1:25600	2.786	2.925	6.708	1.938

*The PI ratio was calculated using the formula PI (%) = [1- (OD_492nm_ of HpSA sample / OD_492nm_ of negative HpSA)] × 100%. HpSA: H. pylori Stool Antigen.

In the present study, the standard procedure of the newly developed rPSA D15-cELISA and rCag11-cELISA assays system were designed as follows: (1) The 96-well ELISA plate was coated with an optimal amount (1μg/100 μL /well) of recombinant (rPSA D15/rCag11) antigens except for Blank (Blank: no reagent) and incubated overnight at 4°C. (2) The plate was then blocked with 100 μl of original concentration Blockace at RT for 1 h. (3) After washed the plate 3x with PBST, a pre-incubation step was performed using 100 μL per well of testing mixtures that included a 1000x diluted stool sample along with anti-rPSA D15/rCag11 polyclonal antibody (1:1 volume/volume), added to all wells except the Blank (which received PBS-Tween), followed by incubation at 37°C for 1 hour. (4) Following three washes with PBST, 100 μl/well of optimally diluted (5,000x) goat anti-rabbit mAb-HRP Conjugate was added to all wells except for Blank (Blank: PBS-Tween) and incubated at 37 °C for 1h. Followed by OPD (100 μL/well) was added and incubated for optimal times (30 minutes) at RT in the dark. (5) Finally, the colorimetric reaction was halted with 100 μL of 3 M H2SO4 per well, and the optical density at 492 nm was measured using an automated ELISA plate reader (Bio-Rad, USA). The results were converted into percent inhibition (PI%). The calculation for percent inhibition (PI%) of the test sample is as follows: (1– OD492nm of the unknown sample ÷ OD492nm of the negative sample mean) X 100%.

The cut-off value for the newly established rPSA D15-cELISA and rCag11-ELISA was determined to be 20.54% (which is 6.45% + 3 times 4.69%). Consequently, *H*. *pylori* stool sample results yielding PI ≥ 20.54% were interpreted as positive, while those with PI < 20.54% were considered negative (Fig 4).

### 3.4. Determination of analytical sensitivity and specificity of the developed cELISA assay

To evaluate the analytical sensitivity of the rPSA D15-cELISA and rCag11-cELISA assays developed in this study, twofold serial dilutions (neat, 1:10, 1:100, 1:1000, 1:2000, 1:4000, 1:8000, 1:16000, 1:32000, 1:64000, 1:128000, 1:256000) of 2 H. *pylori* positive stool antigen samples (verified using a commercial *H*. *pylori* antigen test immunochromatography kit) were tested using rPSA D15-cELISA and rCag11-cELISA. The results indicated that the rPSA D15-cELISA was positive at dilutions ranging from neat to 1:16000, while the rCag11-cELISA showed positive results from neat to 1:32000 (Fig 5B). Consequently, the sensitivity limits for the rPSA-D1-ELISA and rCag11-cELISA for detecting positive *H*. *pylori* stool antigens were determined to be 0.186μg/ml and 0.094μg/ml, respectively, based on the standard antigen concentration (refer to Fig 5A).

Moreover, the analysis of analytical specificity using *H*. *pylori* negative stool antigen (human stool spiked with ESKAPE bacterial whole cell antigen) demonstrated that the percentage inhibition (PI %) values for *H*. *pylori* negative human stool spiked with ESKAPE bacteria ranged from 1.044% to 12.983%, except for *Enterobacter species*, which recorded a PI of 30.97% in the rPSA D15-cELISA. These values fall below the cut-off threshold for both cELISA tests (Fig 6), indicating that the rPSA D15-cELISA and rCag11-cELISA assays have a strong analytical specificity for detecting *H*. *pylori* antigens in stool samples. However, the rPSA D15-cELISA yielded a positive result of 30.97% with *Enterobacter species*, suggesting that it might share a similar protective surface antigen epitope structure with *H*. *pylori* strains.

### 3.5. Evaluation of diagnostic sensitivity and specificity of the new developed cELISA assay

To determine the diagnostic sensitivity of the newly developed rPSA D15-cELISA and rCag11-ELISA, 60 clinical *H*. *pylori (30* positive and 30 negative) stool antigen samples (confirmed by the commercial *H*. *pylori* antigen test immunochromatography kit) were assayed using rPSA D15-cELISA and rCag11-ELISA assay. The findings revealed 3 false positives and 2 false negatives with the rCag11-cELISA (Fig 7A1), while the rPSA D15-cELISA showed 3 false positives and 3 false negatives (Fig 7B1). Additionally, the results from both assays were evaluated using a Receiver Operating Characteristic (ROC) curve analysis. This analysis indicated that the rCag11-cELISA demonstrated the best correlation of diagnostic sensitivity of 93.33% (95% CI 78.68–98.82) and specificity of 90% (95% CI 74.38–96.54) with an optimal cut-off of ≥ 24.16% for positive results and <24.16% for negative results (Fig 7A2). While the rPSA D15-cELISA assays showed a strong correlation between sensitivity and specificity of 90% (95% CI 74.38–96.54) at a cut-off of ≥20.80 for positive samples and <20.80 for negative samples (Fig 7B2), as detailed in Table 3. The Area Under the Curve (AUC) values for the rCag11-cELISA and rPSA D15-cELISA assays were recorded at 0.986 and 0.955, respectively, with a P-value of <0.0001, which is below the significance level of alpha = 0.05. These AUC values, which are close to 1.0, indicate that both the rPSA D15-cELISA and rCag11-cELISA assays are highly effective diagnostic tools for detecting *H*. *pylori* antigens in human stool samples.

**Table 3:** Diagnostic performance of the newly developed rPSA D15-cELISA and rCag11-cELISA assays.

Cutoff value	Assay	Sensitivity (%)	Specificity (%)	Accuracy (%)	NPV (%)	PPV (%)	FPV	FNV	AUC
ROC curve (24.16%)	rCag11-cELIS	93.33	90.00	91.67	93.1	90.32	9.33	0.07	0.986
ROC curve (20.80)	rPSA D15-cELIS	90.00	90.00	90.00	90.00	90.00	9.00	0.11	0.955

*Diagnostic performance calculated using https://www.medcalc.org/calc/diagnostic_test.php

### 3.6. Determination of the accuracy and repeatability of the newly developed cELISA assay

To evaluate the precision and reproducibility of the developed cELISA, three *H*. *pylori* stool antigen samples were tested in a single plate with triplicate using the developed rPSA D15-cELISA and rCag11-cELISA assays. As a result, the intra-assay coefficients of variation (CV) of the PI were 8.6% in rPSA D15-cELISA) and 7.83% in rCag11-cELISA assays (Table 4). These results revealed that the CV _intra-assay_ value of the newly developed rPSA D15-cELISA and rCag11-cELISA assay were < 10%, indicating that the two cELISA assays exhibits excellent reproducibility, reliability, accuracy, and could be used for routine detection.

**Table 4:** Reproducibility of the newly developed rPSA D15-cELISA and rCag11-cELISA assays.

Assay	Reproducibility	The mean (X) and (SD) of PI% of three *H*. *pylori* positive stool samples				
		1	2	3	Average X/ SD	CV%
rPSA-cELISA	Intra-assay	43.66/3.22	48.08/2.15	48.91/6.93	46.88/4.1	8.6%
rCag11-cELISA	Intra-assay	29.63/2.88	53.14/3.12	51.72/4.18	44.83/3.39	7.83%

*Intra-assay precision was determined from three repetitions (well to well) of three H. pylori stool sample in the same method.

### 3.7. Comparisons of rPSA D15-cELISA and rCag11-cELISA with available commercial *H*. *pylori* diagnosis kit

To evaluate the clinical applicability of the newly developed rPSA D15-cELISA and rCag11-cELISA, 60 *H*. *pylori* stool antigen (30 positive and 30 negative) clinical samples were tested with both cELISA and a commercial *H*. *pylori* antigen immunochromatography kit. The results of both rPSA D15-cELISA and commercial *H*. *pylori* rapid immunochromatography kit assay methods coincided in 53/60 of the *H*. *pylori* stool antigen samples with an agreement rate of 88.33% (Kappa = 0.766). While rCag11-cELISA and commercial *H*. *pylori* antigen immunochromatography kit methods coincided in 54/60 of the *H*. *pylori* stool antigen samples with an agreement rate of 90% (Kappa = 0.799) (Table 5).

**Table 5:** Comparison of commercially available immunochromatography assay with rPSA D15-cELISA and rCag11-cELISA using H. pylori stool antigen positive and negative samples.

Assays		Commercial HpSA rapid test kit		Total (%)	K-coefficient (95%CI)
		Positive	Negative		
rPSA D15-cELISA	Positive	26	4	**30 (50%)**	0.766
	Negative	3	27	**30 (50%)**	
	**Total**	**29 (48.33%)**	**31(51.66%)**	**60 (100%)**	
rCag11-cELISA	Positive	28	3	**31 (51.66%)**	0.799
	Negative	3	26	**29 (48.33%)**	
	**Total**	**31 (51.66%)**	**29(48.33%)**	**60 (100%)**	

Furthermore, the stool samples with inconsistent results between cELISA and commercial immunochromatography reference kit were further tested by culture assay. The results showed that among the seven sample inconsistent results between rPSA D15-cELISA and *H*. *pylori* antibody test reference kit, 4 samples (2 positive and 2 negatives in culture assay) were agreed with the results of rPSA D1-cELISA assay and the remaining 3 samples were negative by culture assay, agreed with the results of *H*. *pylori* antigen test reference kit. While, among the six clinical sample inconsistent results between rCag11-cELISA and *H*. *pylori* antigen test reference kit, 4 samples (2 positive and 2 negatives in culture assay) were agreed with the results of rCag11-cELISA assay and the remaining 2 sample were positive by culture assay, agreed with the results of *H*. *pylori* antigen test reference kit. Collectively, these results indicated that the cELISA has a high agreement with the available commercial H. *pylori* antigen test reference kit, and it is promising for clinical testing.

## 4. Discussions

*H*. *pylori (Hp)* is a major cause of chronic gastritis and gastric cancer, which is responsible for a significant number of cancer-related deaths worldwide [3]. In 2014, the International Agency for Research on Cancer (IARC), which operate under WHO, advocated for effective population-wide screening for *H*. *pylori* infection and early eradication therapy to manage *H*. *pylori* infection and the risk of gastric cancer disease [20]. Despite this recommendation, the development of locally validated diagnostic assays and mass-screening is not a common practiced in many countries due to diagnostic kit and related resource limitations. Instead, this approach is typically used in developed countries where *H*. *pylori* infection and gastric cancer are less prevalent [21].

Nowadays, several non-invasive tests including enzyme-linked immunosorbent assay, immunochromatographic assay, and chemiluminescence immunoassay have been developed to diagnose and manage *H*. *pylori* infection. Despite being very limited available in some laboratories and totally absent in others, because of their expensiveness, inconsistence specificity, and sensitivity, have hindered their use in many developing countries where *H*. *pylori* infection is prevalent [22]. In search of rapid, reliable, affordable way to diagnosis of *H*. *pylori* infection, development of serological and stool test based immunodiagnostics assay have been considered an attractive option [23]. Recent studies have focused on novel *H*. *pylori* proteins that have emerged as potential immunodominant antigens with some gene design, which could be used to develop new diagnostic tests and improve the accuracy of *H*. *pylori* detection [8].

Therefore, this study aimed to develop a novel simple, rapid, reliable and accurate *H*. *pylori* stool antigen based competitive enzyme-linked immunosorbent assay (HpSA-cELISA) using a novel immunodominant recombinant (rPSA D15 and rCag11) antigens for the detection of *H*. *pylori* infections. Toward this aim, in this study for ant-rPSA D15 and anti-rCag11 antibody production, 4 rabbits were immunized with the purified rPSA D15 and rCag11 and PBS. The result showed that rabbits immunized with rPSA D15 and rCag11 developed a strong anti-rPSA D15 and anti-rCag11 antibodies immune response, as measured by iELISA. The levels of anti-rPSA D15 and anti-rCag11 antibodies increased rapidly in the first 14–28 days and then stabilized at a high level until the end of the 56-day period. In contrast, rabbits that were not immunized with antigens (pre-immunized and PBS-immunized) did not produce any antibodies against rPSA D15 and rCag11 antigens, indicating that the immunization was effective in inducing a targeted immune response against the purified immunodominant *H*. *pylori* antigens (Fig 1). The results suggest that both the expressed and purified rPSA D15 and rCag11 antigens have a high potential to stimulate a strong immune response in rabbits, as evidenced by a significant increase in anti-rPSA D15 and anti-rCag11 antibody titers with statistically significant difference (p < 0.001) compared to the pre-immunized and PBS-immunized rabbits. The anti-rPSA D15 and anti-rCag11 antibodies generated in this study were used for the first time as a competitive antibody to develop a novel rCag11-cELISA and rPSA D15-cELISA assay for detecting *H*. *pylori* stool antigen in clinical stool sample.

**Fig 2:**
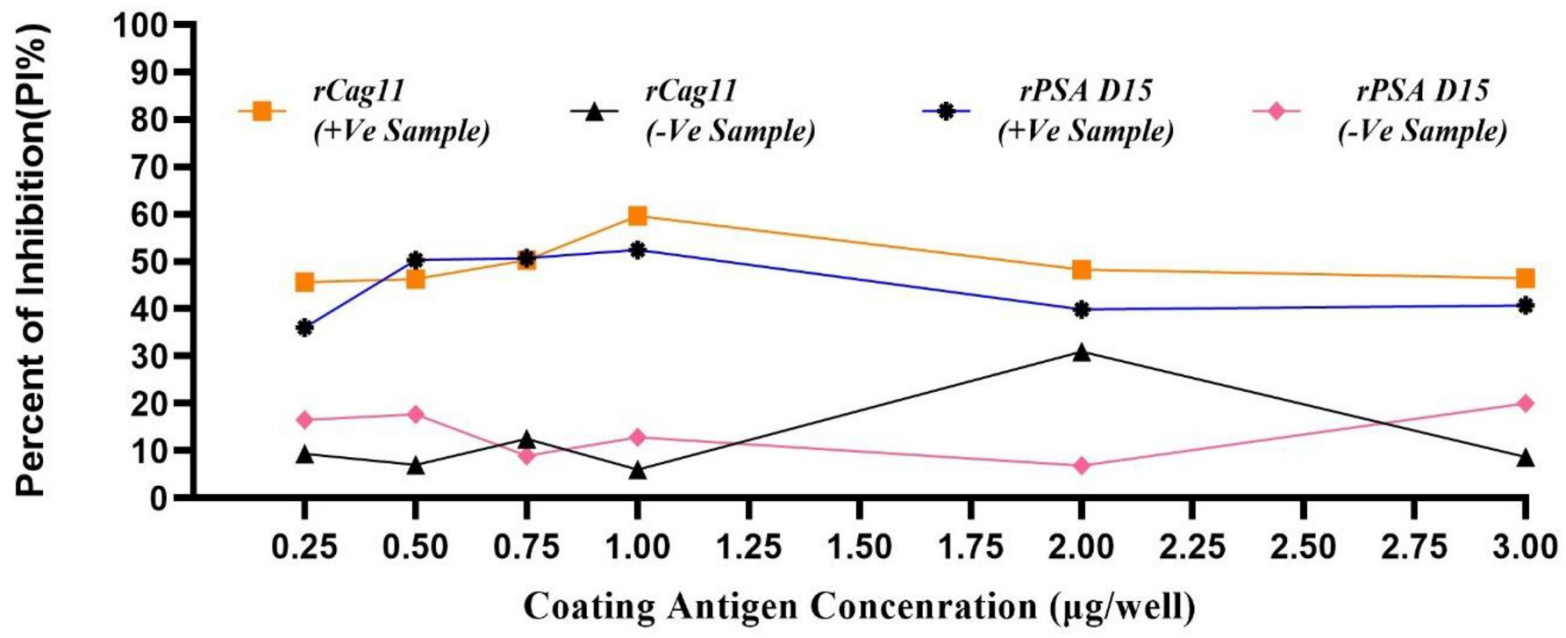
Optimization of coated antigen at different concentration using *H*. *pylori* positive stool antigen (HpSA ^+^Ve), *H*. *pylori* negative stool antigen (HpSA ^-^Ve) samples.

**Fig 3:**
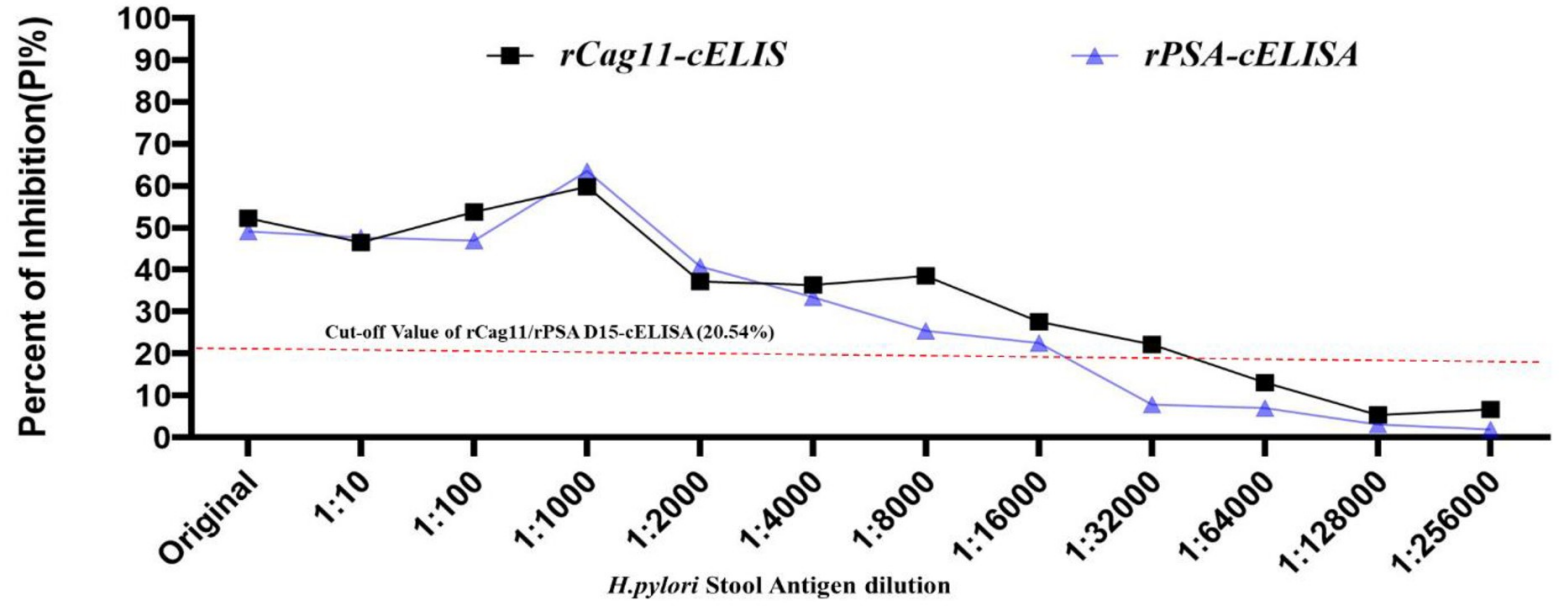
Optimization of the *H*. *pylori* stool antigen sample dilution using rPSA D15-cELISA and rCag11-cELISA assays.

During developing a diagnostic test, it is crucial to establish optimal performance conditions for every step in the protocol. Therefore, optimizing, validating, and standardizing (OVS) the parameters of the newly developed cELISA parameters mainly antigen concentration, serum and the enzyme-conjugate dilution, incubation and color development time are extremely important and necessary to ensure accurate, reproducible, and precise results [24]. Accordingly, in this study, the coated antigen concentration of 1000ng/ml, the anti-rPSA D15 and anti-rCag11 antibodies dilution of 1:1000, the HRP-labelled antibody dilution of 1:5000 and test stool sample diluted at 1:1000 with 1hr incubation and 15 minutes color development cELISA system showed optimum detection level even in the negative control which indicating these aforementioned parameters were present in optimum condition to provide accurate and reliable detection result (Figs 2 & 3).

**Fig 4:**
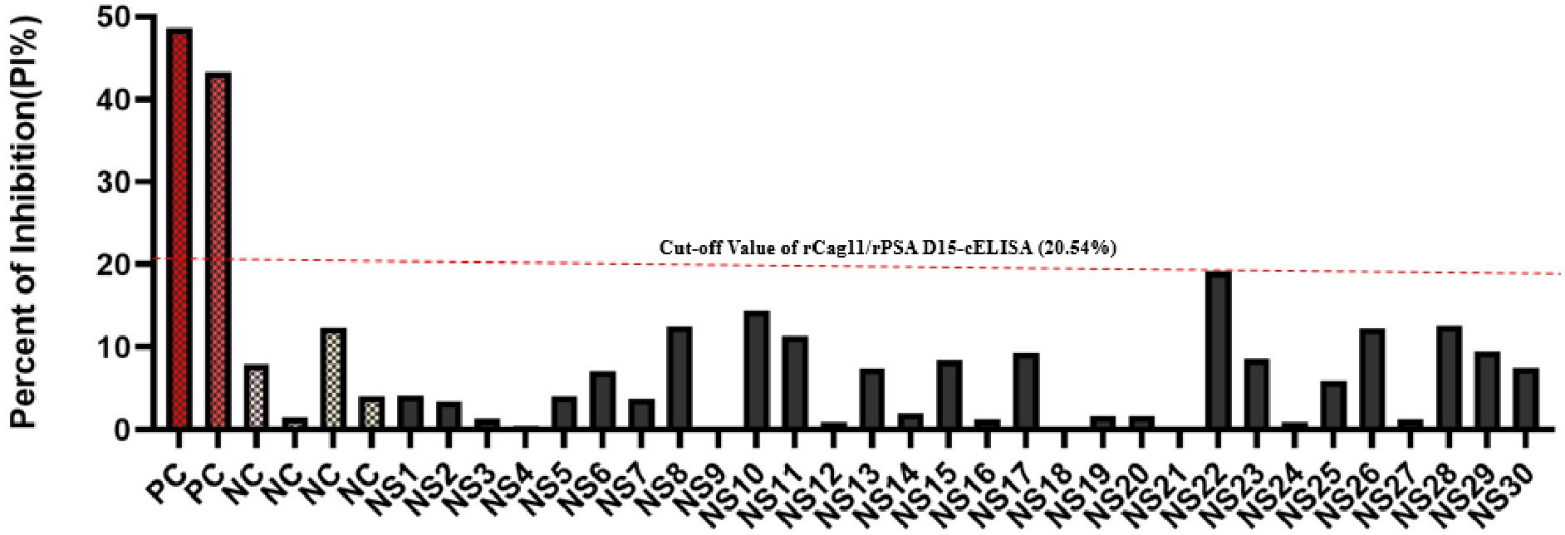
Determination of the cut-off value for rPSA D15-cELISA and rCag11-cELISA assays using rPSA D15/rCag11 (PC), PBS (NC) and *H*. *pylori* negative stool antigen samples (NS1-NS30). The cut-off value (20.54%) is represented by dashed lines(red).

**Fig 5:**
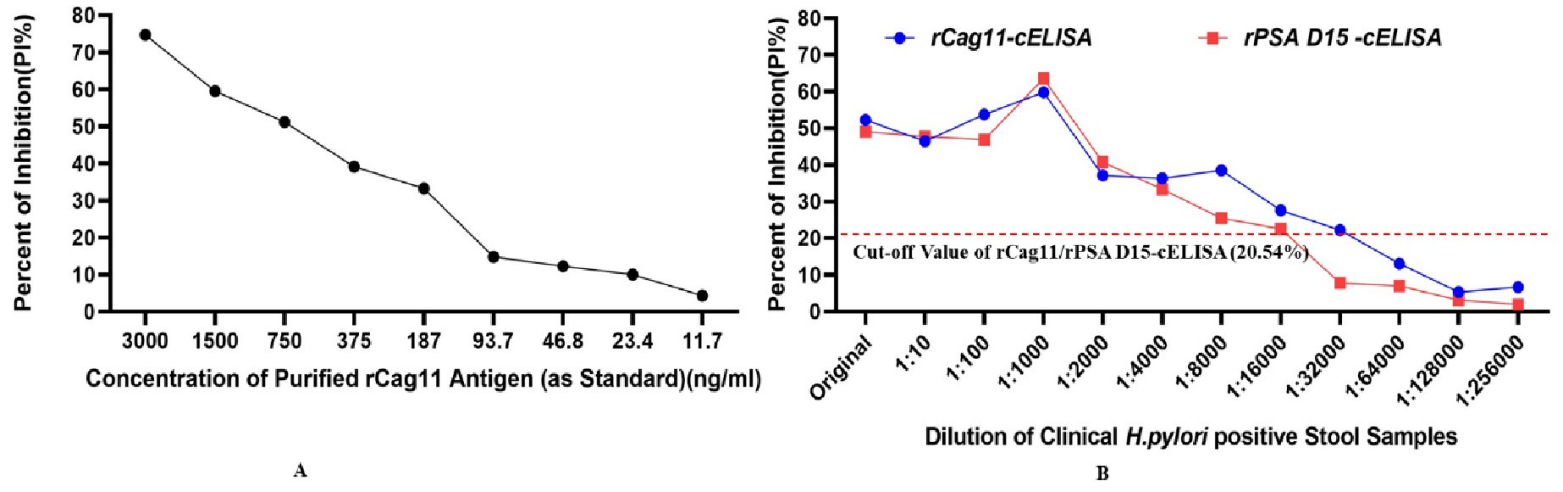
Analytical sensitivity analysis of the developed rPSA D15-cELISA and rCag11-CELISA assay. Determination of the smallest amount of *H*. *pylori* stool antigen detected by the newly developed cELISA assays in μg/ml. An PI% ≥ 20.54% were considered to indicate a positive reaction.

**Fig 6:**
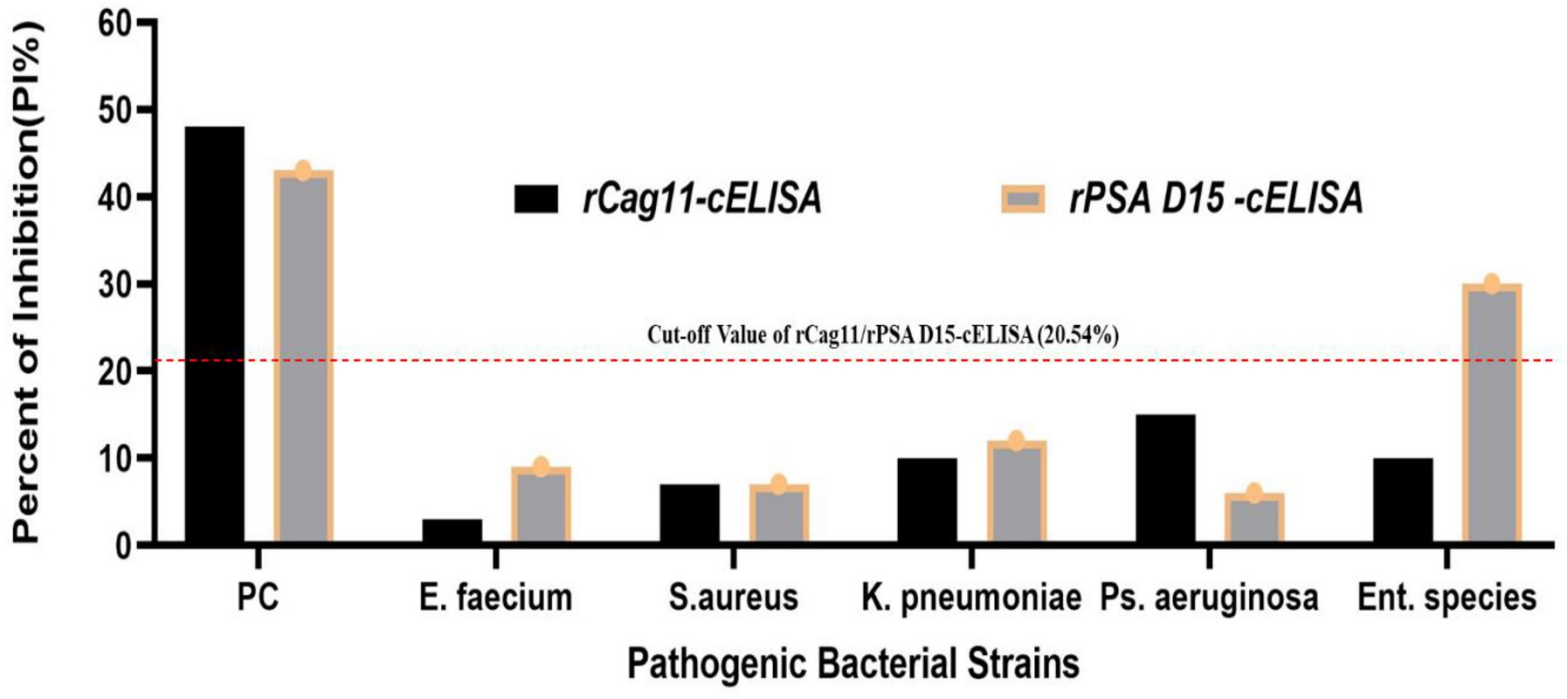
Analytical specificity analysis of the developed rPSA D15-cELISA and rCag11-cELISA assay.

**Fig 7:**
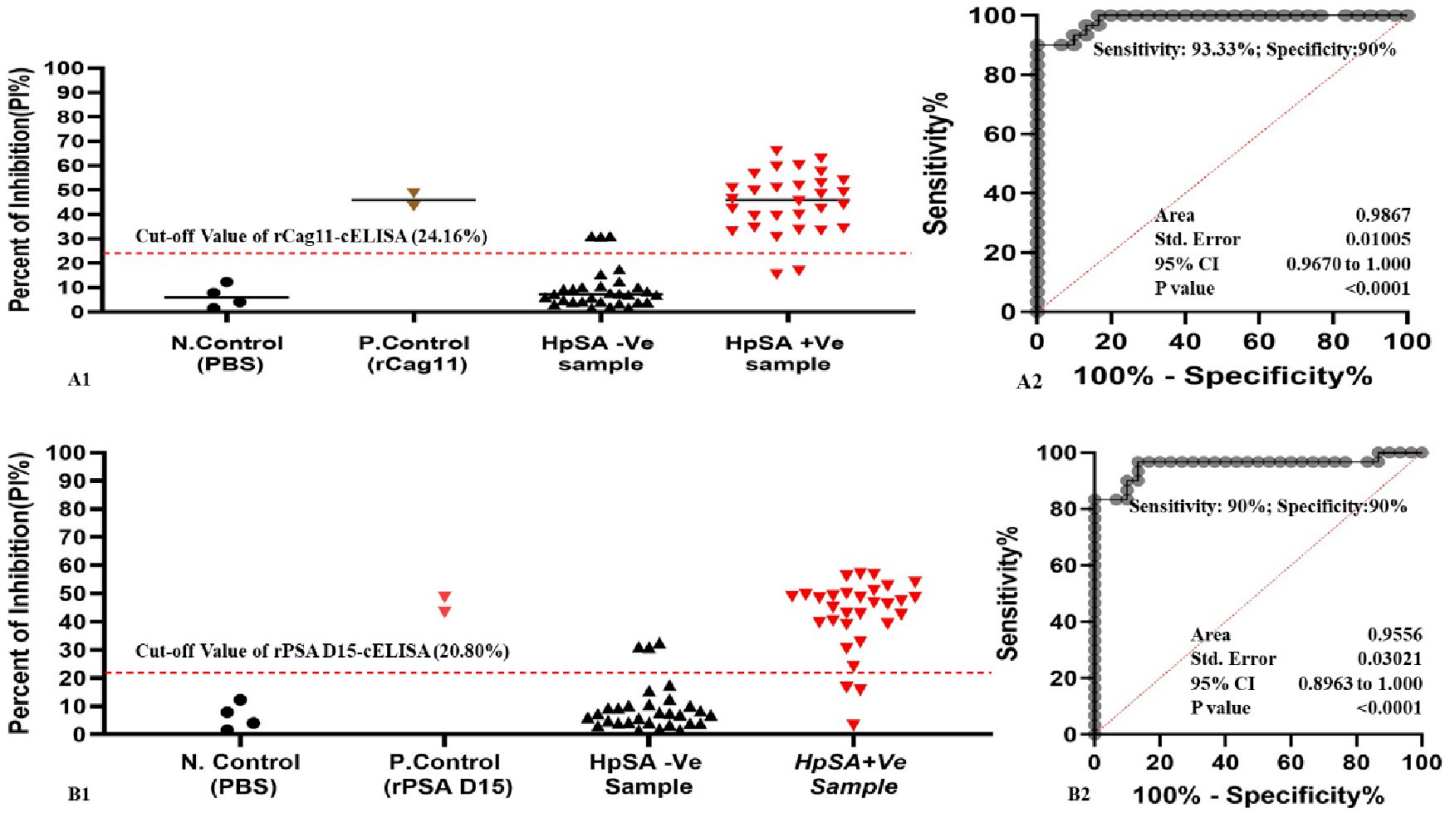
Diagnostic sensitivity and specificity analysis of the developed rCag11-cELISA and rPSA D15-cELISA. Distribution of the PI (%) values reflecting the presence/absence of *H*. *pylori* antigen in the *H*. *pylori* positive and negative stool antigen samples using rCag11-cELISA (A1) and rPSA D15-CELISA assay (B1); ROC curves analysis for diagnostic sensitivity and specificity of rCag11-cELISA (A2) and rPSA D15-CELISA assay (B2).

In the present study, based on the optimized condition novel *H*. *pylori* rPSA D15-cELISA and *r*Cag11-cELISA) assay were developed and validated. The cut-off value for differentiate the positive and negative result of the newly developed rPSA D15-cELISA and rCag11-cELISA was 2.54%PI (Fig 4). In the present study, 60 clinical stool samples were analysed using the newly developed cELISA assays, and the newly developed rPSA D15-cELISA and rCag11-cELISA assays revealed a high overall performance with sensitivity of 90% and 93.33%, specificity of 90% and accuracy of 90% and 91.67% (Table 3) with a limit of detection (LOD) of 0.186μg/ml and 0.094 μg/ml, respectively (Fig 5), suggesting both rPSA D15-cELISA and rCag11-cELISA were the most reliable test for the diagnosis of *H*. *pylori* infection in stool sample. Regarding cross-reactivity, both rPSA D15-cELISA and rCag11-cELISA assays were not showed cross-reactivity with antigenic proteins of non-*H*. *pylori* bacterial strains evaluated (Fig 6) except for one *Enterobacter species*, which indicates that these cELISA assays developed in this study could specifically detect *H*. *pylori* antigen in stool sample. Furthermore, based on the statistical analysis of data generated from clinical *H*. *pylori* stool antigen sample tested using rPSA D15-cELISA and rCag11-cELISA assay revealed that rPSA D15-cELISA and rCag11-cELISA shows AUC of 0.9556 (95% CI = 0.896–1.000) and 0.986 (95% CI = 0.967–1.000) respectively (Fig 7). Therefore, we can conclude that these results convince that both rPSA D15-cELISA and rCag11-cELISA assays can be used in clinic for quickly and accurately detection of *H*. *pylori* infection disease.

Throughout, a year several ELISA (Premier Platinum HpSA Plus test; Meridian Bioscience, USA; Diagnostec *H*. *pylori* antigen EIA kit, Reininghun Diagnostics Biomedical Inc, Taiwan and IDEIA HpStAR, Thermo Fisher Scientific, USA), Immunochromatographic Assay (Wondfo one-step *H*. *pylori* feces test, Guangzhou Wondfo Biotech Co., Ltd., China; ImmunoCard STAT! HpSA, Meridian Diagnostics, USA; Testmate Rapid Pylori antigen test, Wakamoto Pharmaceutical Co., Ltd., Japan; Immunocard ST HpSA, FujiRebio Co., Ltd., Japan; Uni-Gold *HpAT*, Trinity Biotech, Ireland; RAPID Hp StAR, Oxoid Ltd., United Kingdom; Genx H. pylori card test, Genx Bioresearch, Turkey) [20]; and chemiluminescence immunoassay (Liaison H. pylori SA assay, DiaSorin, Stillwater, USA) for the detection of *H*. *pylori*-specific antigen from stool samples have been developed and reported with a specificity range of 87.0–92.4% and sensitivity range of 48.9–92.2%) [25,26].

Despite numerous reports, it has been found that most commercial immunodiagnostic assays for *H*. *pylori* are not only expensive but also exhibit varying specificity and sensitivity when used in different populations across diverse geographical regions [27]. According to previous studies by Khanna and Stone et al., these disparities in detection accuracy are due to the heterogeneity of *H*. *pylori* strains, which can lead to differences in immunogenic epitopes across different parts of the world [28], Therefore, developing immunodiagnostic assay with targeting the most conserved common immunodominant antigens shared globally strains is a crucial strategy, hence in the present study, the rPSA D15-cELISA and rCag11-cELISA assays were developed by targeted stable and conserved immunodominant recombinant (rPSA D15 and rCag11) antigens that are commonly shared in the majority of *H*. *pylori* strains [8], due to that our study showed high reproducibility, sensitivity, specificity and reliable and accuracy to detect *H*. *pylori* in patient using stool sample in comparing to these previously reported diagnostic assays.

Moreover, the variability and reproducibility detection of the rPSA D15-cELISA and rCag11-cELISA assays developed in this study revealed that the reproducibility assays coefficients of variation (CV) were less than 10% (Table 4), indicating that the assays had strong stability (repeatability). Subsequently, to validate their clinical application, the comparative study results of the rPSA D15-cELISA and rCag11-cELISA developed in the present study showed a high agreement (k = 0.766 and 0.799) (Table 5) with the commercially available *H*. *pylori* antigen test immunochromatographic kits, which were currently used in clinical application as a de facto gold standard *H*. *pylori* stool antigen test kit. More importantly, the newly developed novel rPSA D15-cELISA and rCag11-cELISA assay revealed a higher sensitivity and specificity than the clinical available commercial immunochromatography kit by detecting stool antigen of *H*. *pylori* strain. This may be due to that the available commercial immunochromatography kit was developed and validated using their local *H*. *pylori* strain as a source of antigens, which may not be covered all *H*. *pylori* strain antigens found in various geographical regions. While in this study, the rPSA D15-cELISA and rCag11-cELISA assay were developed by targeted the conserved antigen sequence regions of different *H*. *pylori* isolates found in various regions, which enhanced the accuracy and consistent specificity and sensitivity of the developed cELISA kits.

In conclusion, these results indicated that the newly developed rapid, simple, reliable, sensitive and specific rPSA D15-cELISA and rCag11-cELISA assays were a potential reliable and a clinically useful assay for rapid diagnosis and large-scale epidemiological investigation of *H*. *pylori* infection. The assay will be commercially available when field trials in other laboratories and additional regulatory licensure requirements are successfully completed.
